# 三相中空纤维-液相微萃取-高效液相色谱法快速测定烟用香精中9种防腐剂

**DOI:** 10.3724/SP.J.1123.2023.08012

**Published:** 2024-08-08

**Authors:** Ye WANG, Jianchen XIE, Lingjie HUANG, Zhicheng XIA

**Affiliations:** 上海烟草集团有限责任公司质量监督检测站, 上海 200082; Quality Inspection and Test Station, Shanghai Tobacco Group Co. Ltd., Shanghai 200082, China

**Keywords:** 中空纤维-液相微萃取, 高效液相色谱, 防腐剂, 烟用香精, hollow fiber-liquid phase microextraction (HF-LPME), high performance liquid chromatography (HPLC), preservative, tobacco flavor

## Abstract

烟用香精不仅可应用于传统卷烟,其在电子烟、加热卷烟及口含烟等新型烟草制品中均有着广泛应用。烟用香精的含水量较高,为避免香精产品在生产、储存和运输环节发生霉变,往往加入一定量的防腐剂(如山梨酸、苯甲酸及对羟基苯甲酸酯类化合物),以抑制细菌的生长。然而,若超标添加上述防腐剂,亦会对消费者健康带来不良影响。本研究基于三相中空纤维-液相微萃取(3P-HF-LPME)-高效液相色谱法,建立了一种同时测定烟用香精中山梨酸、苯甲酸及7种对羟基苯甲酸酯类防腐剂的分析方法。对仪器参数和HF-LPME条件进行优化,采用Agilent Poroshell 120 EC-C18色谱柱(100 mm×3 mm, 2.7 μm)对9种防腐剂进行分离,以甲醇、0.02 mol/L乙酸铵水溶液(含0.5%乙酸)和乙腈作为流动相进行梯度洗脱,检测波长为226 nm(苯甲酸)和258 nm(山梨酸和7种对羟基苯甲酸酯类化合物)。以二己醚为萃取溶剂,15 mL样品溶液(pH 4)为样品相,氢氧化钠水溶液(pH 12)为接受相,在800 r/min下搅拌萃取30 min。实验结果表明,9种目标分析物在各自的线性范围内线性关系良好,相关系数(*r*)≥0.9967,检出限为0.02~0.07 mg/kg,定量限为0.08~0.24 mg/kg,富集倍数为30.6~91.1,在3个加标水平下的回收率为82.2%~115.7%,相对标准偏差(*n*=5)≤15.0%。该方法简单高效,准确可靠,灵敏度高,适用于烟用香精中防腐剂的快速筛查。

## Abstract (en)

Tobacco flavors are extensively utilized in traditional tobacco products, electronic nicotine, heated tobacco products, and snuff. To inhibit fungal growth arising from high moisture content, preservatives such as benzoic acid (BA), sorbic acid (SA), and parabens are often incorporated into tobacco flavors. Nonetheless, consuming preservatives beyond safety thresholds may pose health risks. Therefore, analytical determination of these preservatives is crucial for both quality assurance and consumer protection. For example, BA and SA can induce adverse reactions in susceptible individuals, including asthma, urticaria, metabolic acidosis, and convulsions. Parabens, because of their endocrine activity, are classified as endocrine-disrupting chemicals. Despite extensive research, the concurrent quantification of trace-level hydrophilic (BA and SA) and hydrophobic (methylparaben, ethylparaben, isopropylparaben, propylparaben, butylparaben, isobutylparaben, and benzylparaben) preservatives in tobacco flavors remains challenging. Traditional liquid phase extraction coupled with high performance liquid chromatography (HPLC) often results in high false positive rates and inadequate sensitivity. In contrast, tandem mass spectrometry offers high sensitivity and specificity; however, its widespread application is limited by laborious sample preparation and significant operational costs. Therefore, it is crucial to establish a fast and sensitive sample pretreatment and analysis method for the nine preservatives in tobacco flavors. In this study, a method for the simultaneous determination of the nine preservatives (SA, BA and seven parabens) in tobacco flavor was established based on three phase-hollow fiber-liquid phase microextraction (3P-HF-LPME) technology combined with HPLC. To obtain the optimal pretreatment conditions, extraction solvent type, sample phase pH, acceptor phase pH, sample phase volume, extraction time, and mass fraction of sodium chloride, were examined. Additionally, the HPLC parameters, including UV detection wavelength and mobile phase composition, were refined. The optimal extraction conditions were as follows: dihexyl ether was used as extraction solvent, 15 mL sample solution (pH 4) was used as sample phase, sodium hydroxide aqueous solution (pH 12) was used as acceptor phase, and the extraction was carried out at 800 r/min for 30 min. Chromatographic separation was accomplished using an Agilent Poroshell 120 EC-C18 column (100 mm×3 mm, 2.7 μm) and a mobile phase comprising methanol, 0.02 mol/L ammonium acetate aqueous solution (containing 0.5% acetic acid), and acetonitrile for gradient elution. Under the optimized conditions, the nine target analytes showed good linear relationships in their respective linear ranges, the correlation coefficients (*r*) were ≥0.9967, limits of detection (LODs) and quantification (LOQs) were 0.02-0.07 mg/kg and 0.08-0.24 mg/kg, respectively. Under two spiked levels, the enrichment factors (EFs) and extraction recoveries (ERs) of the nine target analytes were 30.6-91.1 and 6.1%-18.2%, respectively. The recoveries of the nine target analytes ranged from 82.2% to 115.7% and the relative standard deviations (RSDs) (*n*=5) were less than 14.5% at low, medium and high levels. The developed method is straightforward, precise, sensitive, and well-suited for the rapid screening of preservatives in tobacco flavor samples.

防腐剂是食品、化妆品等工业品的关键添加成分,其能够阻止微生物生长,对产品的质量安全起到重要作用^[1]^。作为食品添加剂和防腐剂,苯甲酸(BA)和山梨酸(SA)已被广泛用于延长产品的保质期^[2]^。对羟基苯甲酸酯类化合物也是食品、化妆品等行业中常用的防腐剂,其可通过破坏细胞膜和胞内蛋白质来改变微生物细胞的酶活性,从而起到防腐的作用^[3,4]^。上述防腐剂通常被认为是安全的,但若超标使用也会带来安全隐患,如过量摄入BA和SA可能会导致代谢性酸中毒、痉挛和过敏等一系列不良反应^[5]^。有研究^[6]^表明,对羟基苯甲酸酯类化合物具有雌激素活性,可能会干扰人体内分泌系统,并且其雌激素活性会随链长的增加而增加。因此在2014年后,长碳链对羟基苯甲酸酯类化合物的使用逐步受到各国法律的限制。不同于日化用品,烟用香精中的防腐剂可随消费者的抽吸或口含行为直接进入体内,对消费者的健康存在潜在威胁。目前国内烟草制品防腐剂行业标准(YC/T 423-2011)^[7]^采用液液萃取作为样品前处理方法,其灵敏度和选择性不高,并且该标准中未包含对长碳链对羟基苯甲酸酯类化合物的检测。为了对烟用香精中防腐剂的使用情况进行有效监管,建立复杂基质中SA、BA及对羟基苯甲酸酯类化合物的简单、快速样品前处理方法及灵敏的分析方法尤为重要。

应用于香精中的防腐剂检测方法主要有气相色谱-质谱法(GC-MS)^[8]^、高效液相色谱法(HPLC)^[9,10]^、超临界流体色谱-串联质谱法(SFC-MS/MS)^[11]^和高效液相色谱-串联质谱法(HPLC-MS/MS)^[12,13]^;应用于香精中的防腐剂前处理方法主要有液液萃取^[9,10,12]^、固相萃取^[8]^和分散固相萃取^[11]^等,其中以液液萃取最为常见,但这些方法普遍存在操作繁琐、专一性差、有机溶剂消耗量大等缺点。一些新型液相微萃取方法,如分散液液微萃取(DLLME)^[14]^,虽具有简单快捷、富集效果好等特点,但由于基质净化能力不足而限制了其进一步的应用。中空纤维-液相微萃取(HF-LPME)是一种以多孔中空纤维为载体的液膜分离技术^[15]^,该技术的萃取和净化过程可一步完成,具有价格低廉、净化效率高等优点,适用于复杂基质中痕量分析物的分离与富集^[16⇓-18]^。近年来,HF-LPME技术已被陆续应用于防腐剂检测^[19,20]^领域,但尚未见将其用于烟用香精中SA、BA和对羟基苯甲酸酯类化合物同时测定的相关报道。

针对以上问题,本研究建立了基于三相中空纤维-液相微萃取(3P-HF-LPME)技术结合HPLC同时测定烟用香精中9种防腐剂(SA、BA和7种对羟基苯甲酸酯类化合物)的分析方法。通过对色谱条件和3P-HF-LPME条件进行优化,实现了烟用香精中9种防腐剂的快速、灵敏测定,为烟用香精中防腐剂的有效监管提供了技术支撑。

## 1 实验部分

### 1.1 仪器、试剂与材料

1260 Infinity高效液相色谱仪(美国Agilent公司); S400酸度计(瑞士Mettler Toledo公司);多点位数显温控磁力搅拌仪(上海越众仪器设备有限公司); Milli-Q超纯水仪(美国Millipore公司);微量进样针(瑞士Hamilton公司);聚偏氟乙烯(PVDF)中空纤维(外径800 μm,内径600 μm,孔径0.2 μm)购自天津膜天膜工程技术有限公司。

标准品:SA、BA、对羟基苯甲酸甲酯(MeP)、对羟基苯甲酸乙酯(EtP)、对羟基苯甲酸丙酯(PrP)、对羟基苯甲酸丁酯(BuP)、对羟基苯甲酸异丙酯(iPrP)、对羟基苯甲酸异丁酯(iBuP)和对羟基苯甲酸苄酯(BzP)(纯度均>99%)均购自梯希爱(上海)化成工业发展有限公司。

0.1 mol/L氢氧化钠溶液、盐酸(分析级)、甲醇(HPLC级)、乙腈(HPLC级)和乙酸铵(色谱纯)均购自德国默克公司;甲苯、氯苯、对二甲苯、二己醚、2-辛酮、1-辛醇(纯度均>98%)购自梯希爱(上海)化成工业发展有限公司;氯化钠、丙酮(分析纯)购自国药集团化学试剂有限公司;烟用香精样品由上海牡丹香精香料有限公司提供。实验所用去离子水均由Milli-Q超纯水仪制备。

### 1.2 溶液的配制

#### 1.2.1 混合标准储备液和混合标准工作液

分别准确称取0.1 g标准品(BA、SA、MeP、EtP、PrP、BuP、iPrP和iBuP)及0.225 g BzP标准品于100 mL容量瓶中,用甲醇定容,配制成质量浓度为1 g/L的混合标准储备液,其中BzP的质量浓度为2.25 g/L;准确移取1 mL混合标准储备液于10 mL容量瓶中,用甲醇稀释并定容,配制成质量浓度为100 mg/L的混合标准工作液,其中BzP的质量浓度为225 mg/L,于4 ℃冰箱中保存。

#### 1.2.2 接受相溶液

准确移取1 mL 0.1 mol/L的氢氧化钠溶液于10 mL具有橡胶塞的容量瓶中,用去离子水定容,配制成0.01 mol/L的氢氧化钠水溶液(pH 12),现用现配。

### 1.3 样品制备

水溶性样品:称取2 g样品置于烧杯中,加入一定体积的去离子水,并用2 mol/L的稀盐酸溶液调节pH至4,随后转移至150 mL容量瓶中,用去离子水定容,制备成相应的样品溶液。

不溶于水样品:称取5 g样品置于具塞离心管中,加入约30 mL 30%甲醇水溶液,超声提取5 min后,将上清液转移至50 mL容量瓶中;用30%甲醇水溶液对离心管反复清洗3次,并将上清液转移至容量瓶中,用30%甲醇水溶液定容后,于4 ℃冰箱中保存。使用前,移取20 mL上述溶液于烧杯中,加入一定体积的去离子水,并用2 mol/L稀盐酸溶液调节pH至4,再转移至150 mL容量瓶中,用去离子水定容,制备成相应的样品溶液。

### 1.4 HF-LPME处理步骤

将PVDF中空纤维截取成长度为10 cm的小段,用丙酮浸没,超声处理5 min,自然晾干;之后将PVDF中空纤维浸没在二己醚中,浸泡约1 min,使二己醚充满纤维腔体的内外壁微孔,之后用无纺布擦除多余的有机溶剂。移取15 mL样品溶液于20 mL样品瓶中,放入磁力搅拌子;用微量进样针向中空纤维小段内腔注满氢氧化钠水溶液(pH 12),并使其呈“U”形浸没于样品相中,中空纤维小段两端用穿透了样品瓶硅胶隔垫的一次性注射器针头固定(图1);随后在25 ℃下以800 r/min的转速搅拌30 min,抽取30 μL接受相进行HPLC分析。

**图1:**
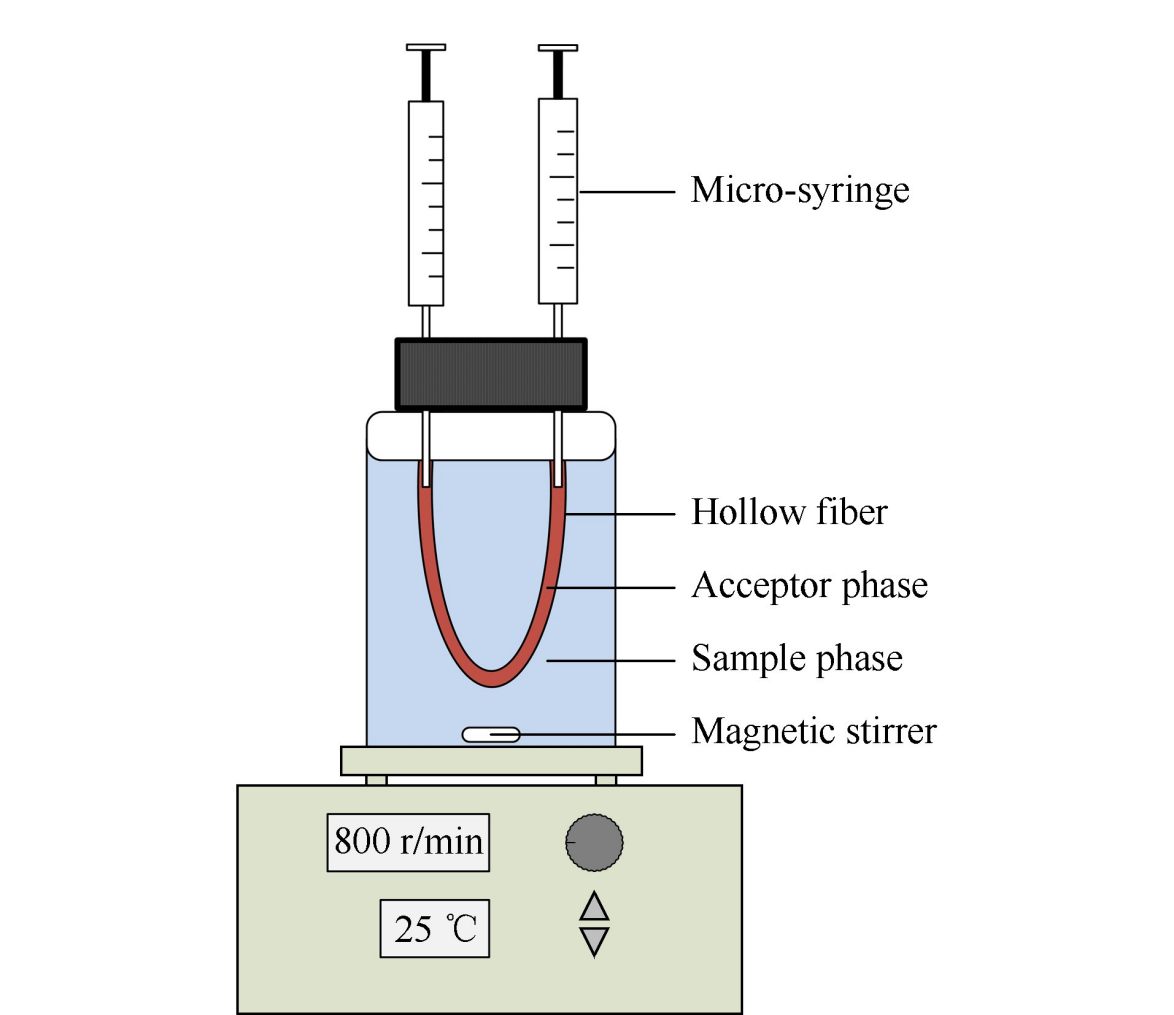
3P-HF-LPME的示意图

### 1.5 富集倍数(EF)与萃取回收率(ER)的计算

EF和ER分别根据公式(1)和公式(2)进行计算:


(1) $$EF=\frac{c_{f}}{c_{0}}$$

(2) $$ER=\frac{c_{f}V_{f}}{c_{0}V_{0}}\times 100\%$$

其中,*c*_0_代表目标分析物在样品相中的原始质量浓度(μg/L), *c*_f_代表目标分析物经3P-HF-LPME萃取后在接受相中的质量浓度(μg/L), *V*_f_是接受相体积(μL), *V*_0_为样品相的初始体积(μL)。

### 1.6 色谱条件

色谱柱:Agilent Poroshell 120 EC-C18(100 mm×3 mm, 2.7 μm);二极管阵列检测器(DAD)扫描范围:200~500 nm;进样量:5 μL;柱温:35 ℃;流动相:A相为甲醇,B相为0.02 mol/L乙酸铵水溶液(含0.5%乙酸), C相为乙腈;流速0.5 mL/min。梯度洗脱程序:0~3.0 min, 70%B, 15%C; 3.0~16.0 min, 70%B~50%B, 15%C~35%C;16.0~25.0 min, 50%B, 35%C; 25.0~26.0 min, 50%B~70%B, 35%C~15%C。

## 2 结果与讨论

### 2.1 色谱条件优化

#### 2.1.1 检测波长的选择

采用质量浓度为60 mg/L的混合标准工作液(其中BzP的质量浓度为135 mg/L)进行色谱条件优化。使用DAD对混合标准工作液中的各待测组分进行全扫描,结果如图2所示。BA的最大吸收波长为226 nm,其余8种目标分析物的最大吸收波长为258 nm。因此针对9种目标分析物,本实验设定两个最佳检测波长(226 nm和258 nm),其中BA的最大吸收波长易受到低波长段基质的干扰,需对流动相组成进行优化,以确保定量的准确性。

**图2:**
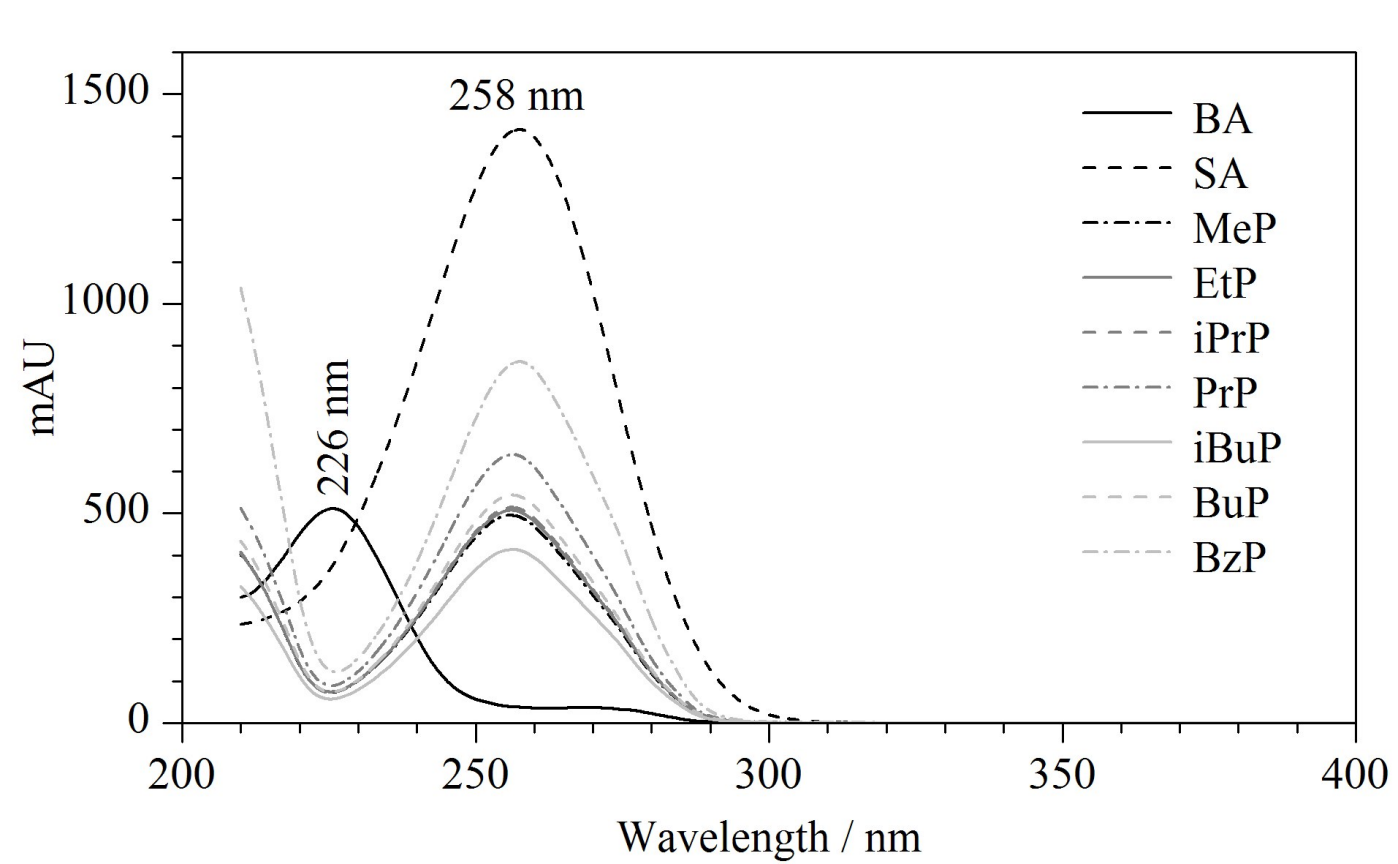
9种目标分析物的紫外吸收图谱

#### 2.1.2 流动相的优化

流动相的组成对于目标分析物的色谱分离影响较大,实验考察了不同流动相组成对9种目标分析物分离效果的影响。实验结果表明,当采用甲醇-乙酸铵水溶液二元流动相时,iBuP、BuP和BzP的分离效果不佳;根据文献[19]改为三元流动相(甲醇-乙酸铵水溶液(含0.5%乙酸)-乙腈)后,iBuP、BuP和BzP在色谱柱上可实现基线分离。经实验考察,乙酸铵水溶液中乙酸的体积分数对BA和SA的色谱分离效果影响较大。如图3a所示,当不添加乙酸时,BA和SA处于部分解离状态,色谱保留较弱,色谱峰形差且无法实现基线分离;在乙酸铵水溶液中添加体积分数为0.02%的乙酸时,BA和SA的色谱峰形可得到明显改善(图3b);BA和SA出峰时间随乙酸体积分数(0.1%、0.5%)的增加而延长(图3c和3d)。由于DAD为通用型检测器,色谱分离前段的目标分析物容易受到样品基质干扰,因此本实验选择在乙酸铵水溶液中添加0.5%乙酸。如图3d所示,在最佳流动相组成条件下,BA和SA的保留时间均>2 min,有助于减少基质峰的干扰,确保定量的准确性。

**图3:**
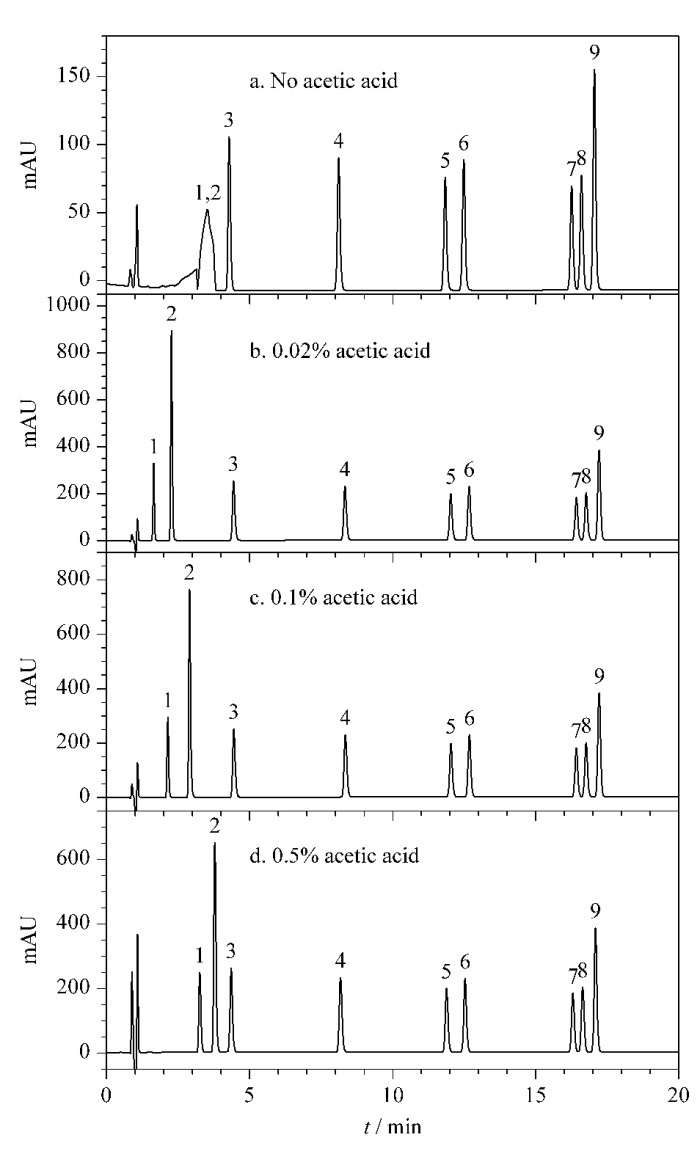
乙酸体积分数对9种目标分析物分离效果的影响

### 2.2 3P-HF-LPME条件优化

#### 2.2.1 萃取溶剂的选择

萃取溶剂是影响HF-LPME萃取效率的最关键因素。本实验选择空白加标样品(60 mg/L,其中BzP加标水平为135 mg/L)进行HF-LPME条件的优化。根据文献[18]中萃取溶剂的选择原则,本文重点考察了甲苯、1-辛醇、二己醚、氯苯、二辛酮5种萃取溶剂对9种目标分析物富集倍数的影响。实验结果表明,极性较强的2-辛酮对BA和SA的萃取效果较好,但对亲脂性较强的对羟基苯甲酸酯类化合物萃取效果差;1-辛醇和二己醚的萃取效果优于其他萃取溶剂,与1-辛醇相比,二己醚对BA、BuP、iBuP和BzP的萃取效果更好,且萃取回收率分布更为均匀(图4a);对1-辛醇和二己醚的萃取效果差异进行分析,可能是因为二己醚的极性适中且黏度更小,更有利于传质。因此,本实验选择二己醚作为3P-HF-LPME的萃取溶剂。

**图4:**
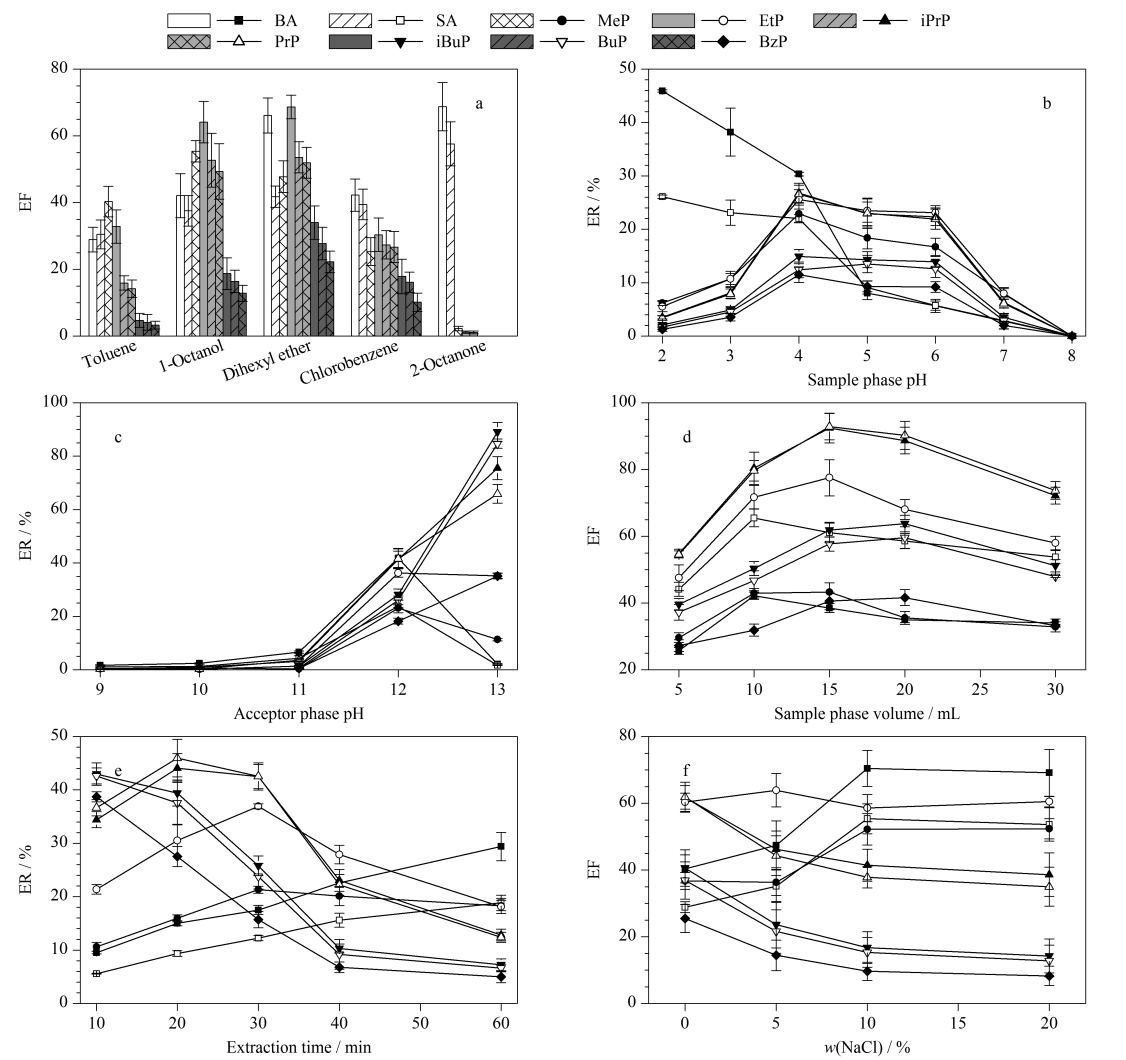
(a)萃取溶剂种类、(b)样品相pH、(c)接受相pH、(d)样品相体积、(e)萃取时间及(f)氯化钠质量分数对9种目标分析物萃取回收率或富集倍数的影响(*n*=3)

#### 2.2.2 样品相/接受相pH的优化

因部分目标分析物为弱酸性且不易溶于水,调节样品相pH至酸性可抑制这些化合物的解离,使其呈分子状态,从而更易被有机液膜萃取;调节接受相pH至碱性则可使这些分析物处于解离状态,更有效地被接受相萃取,避免被反萃取至有机相中,从而保证方法的回收率。因此,本实验分别考察了不同pH(2~8)的样品相(接受相pH为12)及不同pH(9~13)的接受相(样品相pH为4)对9种目标分析物萃取回收率的影响。

如图4b所示,当样品相pH为2时,BA和SA的萃取回收率最高,之后二者的萃取回收率随样品相pH的升高而降低;当样品相pH为4时,7种对羟基苯甲酸酯类化合物的萃取回收率最高,且在pH为4~6时基本维持不变,这与文献[21]报道结果相符。因此,综合考虑BA和SA和7种对羟基苯甲酸酯类化合物的萃取回收率,实验选择样品相的最佳pH为4。由图4c可知,当接受相pH从9增加至13时,大部分目标分析物的萃取回收率升高且达到最大;BA、SA、MeP和EtP的萃取回收率在接受相pH为12时达到最高,而在pH为13时反而下降,并且此时BA和SA已无法被萃取。因此实验选择接受相的最佳pH为12。

#### 2.2.3 样品相体积的优化

实验分别考察了样品相体积为5、10、15、20和30 mL时9种目标分析物的富集倍数。如图4d所示,BA和SA的富集倍数在样品相体积为10 mL时达到最大,MeP、EtP、iPrP和PrP的富集倍数在样品相体积为15 mL时达到最大,而iBuP、BuP和BzP的富集倍数在样品相体积为20 mL时达到最大。综合考虑目标分析物的富集倍数及萃取平衡时间,最终确定样品相体积为15 mL。

#### 2.2.4 萃取时间的优化

萃取时间同样是影响3P-HF-LPME萃取效率的重要因素,实验考察了不同萃取时间(10、20、30、40和60 min)对9种目标分析物萃取效率的影响。实验结果表明,BA和SA的萃取回收率随萃取时间的增加而持续升高;MeP和EtP的萃取回收率在萃取时间为30 min时达到最大,iPrP和PrP的萃取回收率在萃取时间为20 min时达到最大;而对于iBuP、BuP和BzP,萃取时间越短,三者的萃取回收率越高(图4e)。当萃取时间增加至40 min时,大部分长碳链的对羟基苯甲酸酯类化合物(iBuP、BuP和BzP)会发生反萃取现象,并被截留在有机液膜中,该结果与文献[19]报道一致。为兼顾9种目标分析物的萃取效果,最终选择萃取时间为30 min。

#### 2.2.5 离子强度的优化

本研究还考察了不同质量分数(0、5%、10%、20%)的氯化钠对9种目标分析物富集倍数的影响。结果如图4f所示,随着样品相中氯化钠质量分数的增加,BA、SA和MeP的富集倍数有所提升,但其余目标分析物的富集倍数无明显变化甚至有所降低,因此本实验选择不添加氯化钠。

### 2.3 方法学考察

#### 2.3.1 线性关系、检出限、定量限和富集倍数

按照1.3节步骤获得空白样品溶液,加入不同体积的混合标准工作液,配制成系列质量浓度(2、10、20、50、100、200、500、1000和2000 μg/L,其中BzP的质量浓度分别为4.5、22.5、45、112.5、225、450、1125和2250 μg/L)的基质匹配混合标准工作液。在最佳实验条件下进行检测,以9种目标分析物的峰面积(*y*)对质量浓度(*x*, μg/L)绘制标准工作曲线。以3倍信噪比(*S/N*)和10倍*S/N*所对应的含量作为检出限(LOD)和定量限(LOQ)。结果如表1所示,9种目标分析物在各自的线性范围内线性关系良好,相关系数(*r*)均≥0.9967, LOD为0.02~0.07 mg/kg, LOQ为0.08~0.24 mg/kg。在空白样品溶液中分别添加两个水平(3.75 mg/kg和37.5 mg/kg,其中BzP的加标水平分别为8.44 mg/kg和84.4 mg/kg)的混合标准工作液,在最佳实验条件下计算各目标分析物的富集倍数和萃取回收率。结果表明,在两个加标水平下9种目标分析物的富集倍数为30.6~91.1,萃取回收率为6.1%~18.2%,说明本方法的灵敏度高,富集效果明显。

**表1:** 9种目标分析物的线性范围、相关系数、检出限、定量限、富集倍数及萃取回收率(*n*=5)

Compound	Linear range/ (μg/L)	r	LOD/ (mg/kg)	LOQ/ (mg/kg)	EFs			ERs/%	
					3.75 mg/kg^a^	37.5 mg/kg^b^		3.75 mg/kg^a^	37.5 mg/kg^b^
BA	2.0-2000	0.9997	0.02	0.08	91.1	74.9		18.2	15.0
SA	2.0-2000	0.9967	0.03	0.10	45.5	37.3		9.1	7.5
MeP	2.0-1000	0.9990	0.03	0.11	34.5	32.1		6.9	6.4
EtP	2.0-1000	0.9989	0.04	0.13	56.6	51.1		11.3	10.2
iPrP	2.0-1000	0.9990	0.03	0.10	67.8	55.8		13.6	11.2
PrP	2.0-1000	0.9980	0.03	0.10	68.3	61.4		13.7	12.3
iBuP	2.0-1000	0.9977	0.04	0.13	46.9	41.3		9.4	8.3
BuP	2.0-1000	0.9996	0.04	0.14	38.1	30.6		7.6	6.1
BzP	4.5-2250	0.9997	0.07	0.24	40.6	32.5		8.1	6.5

a, b: spiked levels of BzP were 8.44 mg/kg and 84.4 mg/kg, respectively.

#### 2.3.2 回收率与精密度

在空白样品溶液中添加低、中、高3个水平(0.15、3.75和37.5 mg/kg,其中BzP的加标水平分别为0.34、8.44和84.4 mg/kg)的混合标准工作液,在优化后的实验条件下进行加标回收试验,每个加标水平设置5个平行样品。结果如表2所示,在3个加标水平下9种目标分析物的回收率为82.2%~115.7%,相对标准偏差(RSD)均≤15.0%。实验结果表明,本方法的回收率和精密度良好,能够满足9种防腐剂的实际分析需求。

**表2:** 9种目标分析物的回收率与相对标准偏差(*n*=5)

Compound	0.15 mg/kg^c^			3.75 mg/kg^d^			37.5 mg/kg^e^	
	Recovery/%	RSD/%		Recovery/%	RSD/%		Recovery/%	RSD/%
BA	95.3	5.2		98.1	8.0		90.9	6.2
SA	105.1	7.8		102.3	7.8		102.3	8.0
MeP	97.4	6.9		115.7	9.3		82.2	7.1
EtP	82.6	8.6		86.7	10.6		91.7	6.8
iPrP	82.4	4.9		114.7	9.1		88.5	3.2
PrP	96.5	4.3		112.6	9.7		87.9	3.7
iBuP	97.8	9.8		87.8	8.4		107.6	11.5
BuP	114.9	10.0		95.6	11.2		90.8	10.5
BzP	112.0	15.0		104.7	13.3		93.2	14.5

c, d, e: spiked levels of BzP were 0.34, 8.44 and 84.4 mg/kg, respectively.

### 2.4 实际样品检测

选取10个国内企业生产的典型烟用香精样品(其中4号和10号为不溶于水的样品,其余为水溶性样品),按照1.3节步骤获得样品溶液,之后在优化的实验条件下进行萃取和测定。以其中一个样品为例,对其进行加标(37.5 mg/kg, BzP的加标水平为84.4 mg/kg)。如图5所示,加标样品经3P-HF-LPME处理后,其基质干扰峰明显减少且信号强度明显增强,表明该萃取方法具有一定的基质净化功能且富集效果明显。如表3所示,BA和SA在烟用香精中的添加较为普遍,但10个待测样品中的BA和SA均未超过《食品安全国家标准 食品添加剂使用标准》(GB 2760-2014)^[22]^所规定的最大限量值(2000 mg/kg);有5个样品检出了EtP,且其中一个样品同时检出了MeP,依据GB 2760-2014,二者的最大使用限量均为250 mg/kg,虽有添加但符合使用要求;此外,有一个样品检出了PrP,该防腐剂在GB 2760-2014的许可目录之外,因此需引起相关质检部门的关注。

**图5:**
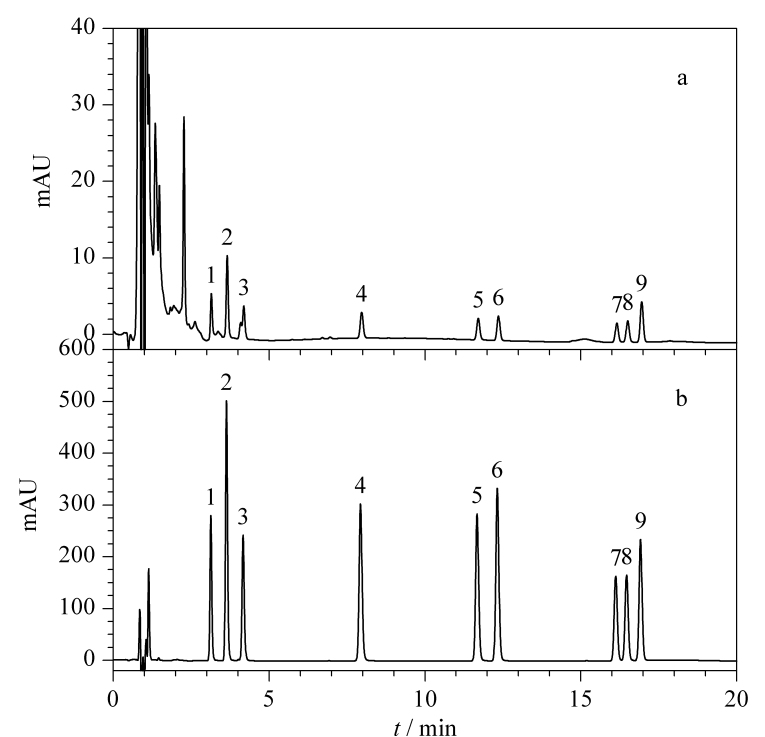
烟用香精加标样品经3P-HF-LPME处理(a)前、(b)后的色谱图

**表3:** 实际样品的检测结果

Sample No.	BA	SA	MeP	EtP	iPrP	PrP	iBuP	BuP	BzP
1	55.4	-	-	0.3	-	0.1	-	-	-
2	21.9	-	-	0.7	-	-	-	-	-
3	-	-	-	-	-	-	-	-	-
4	30.3	1.4	-	2.0	-	-	-	-	-
5	3.5	14.1	-	-	-	-	-	-	-
6	3	12.6	-	-	-	-	-	-	-
7	-	13.7	-	-	-	-	-	-	-
8	9.3	13.9	-	-	-	-	-	-	-
9	19.3	61.5	0.1	0.1	-	-	-	-	-
10	36	5.8	-	0.1	-	-	-	-	-

-: not detected.

### 2.5 与其他方法的比较

将本方法与行业标准及文献报道进行比较,比较结果见表4。结果表明,与采用相同检测器的烟草行业标准(YC/T 423-2011)^[7]^及文献[9⇓-11]相比,本方法的LOD更低。此外,本方法的回收率与其他方法相近,能够满足防腐剂的实际分析要求。

**表4:** 本方法与其他方法的比较

Analytes	Sample pretreatments	Detection technique	Recoveries/ %		LODs/ (mg/kg)		Ref.
BA, SA and four PBs	LLE	HPLC-UV	93.2-	98.5	1.8-	6.0	[7]
BA, SA and four PBs	LLE, UAE	HPLC-UV	97.0-	99.6	0.08-	0.2	[9]
BA, SA, four PBs and other eight analytes	LLE, UAE	UPLC-PDA	93.0-	121.0	0.48-	2.51	[10]
Six PBs	dSPE	SFC-MS/MS	88.3-	106.6	0.03-	0.09	[11]
BA, SA and seven PBs	3P-HF-LPME	HPLC-DAD	82.2-	115.7	0.02-	0.07	this study

PBs: parabens; LLE: liquid-liquid extraction; UAE: ultrasound-assisted extraction; dSPE: dispersive solid-phase extraction; SFC: supercritical fluid chromatography.

## 3 结论

本研究基于3P-HF-LPME技术并结合HPLC,建立了烟用香精中9种防腐剂(SA、BA和7种对羟基苯甲酸酯类化合物)同时测定的方法。利用3P-HF-LPME技术,该方法可一步完成目标分析物的萃取、净化和富集。所建方法操作简单,富集倍数高,同时具有绿色环保、成本低廉的优点,可用于烟用香精中防腐剂的快速筛查。
